# Research perspective in the clinical management of Kawasaki disease

**DOI:** 10.3389/fped.2024.1415941

**Published:** 2024-07-09

**Authors:** Xiong-xiong Yi, Wen-rong Zhang, Dong-mei Wang, Xiu-ping Wang, Fen-xia Zhang

**Affiliations:** ^1^ Department of Pediatrics, Yan’an People’s Hospital, Yan’an, Shaanxi, China; ^2^ Department of Neonatology, Yan’an People’s Hospital, Yan’an, Shaanxi, China

**Keywords:** Kawasaki disease, immunoglobulin, aspirin, pathological mechanism, treatment

## Abstract

This study examines research perspective in the clinical diagnosis, treatment, and prevention of cardiovascular complications in Kawasaki Disease (KD). Starting with an overview of the disease, it introduces KD's clinical manifestations, etiology, epidemiological features, and its impact on the cardiovascular system. Subsequently, the study discusses in detail the diagnostic methods, pathological mechanisms, and treatment strategies for KD, including foundational and emerging approaches such as high-dose intravenous immunoglobulin and aspirin therapy, biologic therapy, and corticosteroid pulse therapy. Additionally, it outlines strategies for preventing cardiovascular complications, including early risk assessment and long-term management. The study also explores the intersection of the COVID-19 pandemic with an increase in KD-like symptoms, emphasizing the need for further studies on the association between SARS-CoV-2 and KD. Lastly, it explores future research directions to enhance understanding of KD and improve patient outcomes and quality of life. This study provides valuable insights into the comprehensive treatment and management of KD and highlights avenues for future research.

## 1 Introduction

Kawasaki Disease (KD) represents an acute, systemic vasculitis predominantly affecting children under five years of age (1, 2). Initially identified by Japanese physician Tomisaku Kawasaki in 1967, the disease is marked by inflammation of medium and small-sized arteries, with particular emphasis on the coronary arteries (3). Beyond impacting the cardiovascular system, KD manifests in the skin, mucous membranes, lymph nodes, and various other organs (1, 2). Its clinical presentation includes persistent fever, rash, conjunctival congestion, alterations in the lips and oral mucosa, changes in the extremities, and cervical lymph node enlargement (1, 2). Without timely intervention, approximately 25% of the afflicted children are at risk of developing critical cardiovascular complications, including coronary artery anomalies (such as dilation or aneurysms), potentially culminating in myocardial infarction, arrhythmias, or sudden death (4).

The etiology of KD remains elusive, although current consensus points towards an interplay between genetic predisposition and environmental triggers (4, 5). Epidemiologically, the disease exhibits a higher prevalence in Asian countries, notably Japan and South Korea, yet it maintains a global presence, impacting numerous families and children internationally (4, 5). Recent epidemiological trends have shown an increase in KD symptoms among children concurrent with the COVID-19 pandemic. Notably, research identified a significant outbreak of Kawasaki-like disease at the core of the SARS-CoV-2 epidemic in Italy (6). These findings suggest the association between SARS-CoV-2 infection and KD-like manifestations, highlighting the pandemic's impact on this vasculitis predominantly affecting young children.

Advancements in the clinical management of KD are of paramount public health significance (1). Prompt and effective interventions can markedly diminish the occurrence of cardiovascular complications and enhance the long-term outlook for the affected children (7). Historically, a regimen of high-dose intravenous immunoglobulin (IVIG) and aspirin has established itself as the cornerstone of KD management, effectively lowering the rate of coronary artery complications (1, 7). Nonetheless, an estimated 10%–20% of pediatric patients demonstrate resistance to initial IVIG therapy, positioning them at an elevated risk for cardiovascular sequelae (8). Consequently, the exploration of novel therapeutic avenues, including corticosteroids, biologic agents, and immunomodulators, has emerged as a focal area of contemporary research (9). Additionally, the evolving comprehension of KD pathophysiological underpinnings holds promise for the formulation of more individualized and targeted therapeutic strategies in the foreseeable future (10).

## 2 KD diagnosis

KD diagnosis relies predominantly on identifying specific clinical signs, as direct diagnostic laboratory tests are currently unavailable (1, 2). The American Heart Association and the Japanese KD Research Committee outline essential clinical criteria for KD diagnosis (1, 2). These criteria necessitate a persistent fever lasting more than five days, unalleviated by medical intervention, coupled with at least four out of the following five principal symptoms (1, 2, 11):

**Bilateral Conjunctival Congestion:** Noted for the absence of purulent discharge, indicating a non-infectious cause of the eye redness.

**Oral and Pharyngeal Manifestations:** These include cracked lips, a strawberry-like appearance of the tongue, or widespread inflammation across the throat area, signaling systemic inflammation.

**Dermatological Rash:** A rash of variable appearance, underlining the systemic nature of the disease.

**Extremity Alterations:** Characterized by swelling and peeling, indicative of peripheral inflammation.

**Cervical Lymphadenopathy:** Defined by lymph node enlargement of at least 1.5 cm in diameter, suggesting an immune response.

Acknowledgment is given to the occurrence of “Incomplete Kawasaki Disease,” where the full spectrum of symptoms may not be present, yet the risk of developing coronary artery anomalies remains (9).

To corroborate the clinical diagnosis, ancillary laboratory tests and imaging studies are employed to exclude differential diagnoses. Elevated white blood cell count, C-reactive protein (CRP), and erythrocyte sedimentation rate (ESR) serve as non-specific markers of systemic inflammation (12). Echocardiography remains paramount in detecting coronary artery irregularities.

Diagnostic capabilities have been significantly enhanced by recent advancements in imaging and molecular biology. Cardiac Magnetic Resonance Imaging and Positron Emission Tomography have demonstrated superior sensitivity and specificity for identifying cardiovascular anomalies over traditional methods. These techniques facilitate the prompt recognition and monitoring of coronary artery changes. Additionally, the exploration of serum biomarkers through molecular biology offers potential for refining KD diagnosis and prognostication. However, the practical application of these novel diagnostic tools still mandates further clinical validation to ascertain their effectiveness and cost-efficiency.

In essence, diagnosing KD is an intricate process that amalgamates clinical observation, laboratory findings, and advanced imaging techniques. Ongoing enhancements in diagnostic technology herald the possibility of more precise KD diagnosis in the future, contributing to earlier therapeutic intervention, minimization of complications, and an improved prognosis for afflicted children.

## 3 KD's pathological mechanism

The etiopathogenesis of KD remains partially elucidated, yet prevailing evidence suggests it results from an intricate interplay between dysregulated immune responses, genetic predispositions, and environmental influences (4, 13, 14). This multifaceted interaction precipitates systemic vasculitis, predominantly affecting the coronary arteries and medium to small-sized vessels.

The COVID-19 pandemic has highlighted the potential for viruses to act as environmental triggers for Kawasaki Disease (KD). The correlation between increased presentations of KD-like symptoms and SARS-CoV-2 infection during the pandemic has been supported by systematic analyses (15). These developments suggest that viral infections, particularly SARS-CoV-2, may initiate inflammatory responses in genetically susceptible populations, thereby contributing to KD pathogenesis.

### 3.1 Immune system dysregulation

KD is characterized by an aberrant activation of the immune system, signified by heightened autoimmune activity (16). Central to this immune dysregulation are T lymphocytes, macrophages, and endothelial cells, each playing pivotal roles in the disease's pathology. The aberrant activation of T cells and macrophages culminates in the excessive release of pro-inflammatory cytokines, such as tumor necrosis factor-alpha (TNF-α) and interleukin-6 (IL-6). These cytokines inflict direct damage on the vascular endothelium, instigating vasculitis. Concurrently, endothelial cell dysfunction augments vascular permeability, exacerbating the inflammatory milieu (17).

### 3.2 Genetic susceptibility

Genetic predisposition is a critical determinant in the susceptibility to KD (13). Investigations have identified specific genetic loci associated with an elevated risk of developing KD (13). Notably, polymorphisms within the HLA class genes, BLK (B lymphocyte kinase), and CD40 have been implicated in augmenting susceptibility to KD. These genetic variations presumably modulate immune regulatory pathways, particularly influencing the functional dynamics of T and B lymphocytes, thereby predisposing individuals to KD (18).

### 3.3 Environmental influences

The precise environmental triggers of KD remain speculative, though hypotheses include viral and bacterial infections, alongside exposure to specific chemicals or toxins. These environmental agents are theorized to initiate inflammatory cascades by engaging the immune system, especially in individuals with a genetic predisposition to KD (18).

### 3.4 Recent advances in understanding KD pathogenesis

Emerging research has yielded significant insights into KD's pathogenesis. For instance, studies exploring the gut microbiome have indicated a correlation between alterations in microbial diversity and KD onset, suggesting a potential role of gut flora in disease development. Furthermore, investigations into microRNAs (miRNAs) in KD patients have unveiled novel aspects of the disease's pathogenic mechanisms (19). These miRNAs are hypothesized to orchestrate inflammatory responses and vascular injury, offering new avenues for understanding KD's molecular underpinnings (19–21).

Recent literature has begun to explore the role of SARS-CoV-2 in modifying the pathogenesis of KD. These studies propose that the virus may influence KD's pathogenesis through its interaction with the host's immune system, potentially leading to KD-like symptoms (22, 23). This emerging evidence lends support to the hypothesis that environmental factors, including viral infections, play a crucial role in the development of KD.

In essence, the pathogenesis of KD involves a complex interplay of immune system dysregulation, genetic predispositions, and environmental triggers. Research highlights the role of aberrant immune activation, genetic factors, and environmental influences, including viral infections like SARS-CoV-2, in triggering inflammatory responses. Advances in understanding microbial diversity and miRNAs further illuminate KD's mechanisms, paving the way for innovative diagnostic and therapeutic strategies.

## 4 Treatment strategies for KD

The therapeutic approach to KD is strategically designed to mitigate the risk of coronary artery abnormalities, relieve symptomatic discomfort, and avert long-term sequelae (16, 24–38). An integrative regimen encompassing pharmacological interventions and supportive care forms the basis of treatment (Table 1) (16, 24–38).

**Table 1:** Treatments in children with KD of clinical trials.

Participants	Type of study	Treatment	Findings	Reference
Children with KD	Randomized controlled trial	Infliximab IVIG (Privigen)	It was safe and well tolerated, reducing fever duration, inflammation markers, left anterior descending coronary artery Z score, and IVIG reaction rates	Tremoulet et al. (16)
Children with KD	Randomized controlled trial	IVIG, TBSF vs. Privigen	Privigen may exhibit a decreased incidence of IVIG resistance and reduced involvement of coronary arteries	Kuo et al. (24)
Children with resistant KD	Randomized controlled trial	Second IVIG vs. Infliximab	Infliximab proves safe, well-tolerated, and effective in treating IVIG-resistant KD, resulting in shorter fever duration, reduced need for additional therapy, milder anemia, and shorter hospital stays compared to a second IVIG infusion	Burns et al. (25)
Children with KD	Randomized controlled trial	IVIG	Efficacy and safety of IVIG administered over 12 h (double speed) were comparable to those administered over 24 h (reference speed)	Fukui et al. (26)
Children with KD	Randomized controlled trial	IVIG and aspirin	A 1 g/kg IVIG dose proves cost-effective with efficacy equivalent to 2 g/kg IVIG, suitable for initial KD therapy	He et al. (27)
Children with KD	Observational study	Aspirin alone or aspirin plus gammaglobulin	Gamma globulin therapy reduced coronary artery lesion incidence and myocardial damage severity from moderate/severe to mild in KD	Haneda et al. (28)
Children with KD	Observational study	High vs. Low doseaspirin	High-dose aspirin may not be ideal for anti-thrombotic treatment, suggesting that low-dose therapy is safe during the acute stage of KD	Akagi et al. (29)
Children with KD	Observational study	Infliximab	Infliximab was well tolerated and effective in treating acute KD refractory to conventional therapies	Miura et al. (30)
Children with KD	Randomized controlled trial	Infliximab vs. IVIG	Infliximab improved defervescence rate within 48 h and time to defervescence compared to standard therapy, and was well tolerated in IVIG-refractory KD	Mori et al. (31)
Children with KD	Randomized controlled trial	Infliximab and IVIG	Treatment of KD patients with infliximab doesn't negatively impact tolerogenic myeloid DC generation or T cell regulation and memory development	Burns et al. (32)
Children with KD	Retrospective study	Infliximab vs. IVIG	In IVIG-resistant KD, initial infliximab re-treatment led to faster fever resolution and reduced hospitalization compared to IVIG, with similar coronary artery outcomes and adverse events	Son et al. (8)
Children with KD	Randomized controlled trial	IVMP + IVIG or IVIG alone	IVMP + IVIG therapy proves safe and effective for predicted refractory KD children	Ogata et al. (33)
Children with KD	Randomized controlled trial	Pulsed corticosteroid therapy (IVMP)	Pulsed corticosteroid therapy for primary treatment of KD	Newburger et al. (34)
Children with KD	Observational study	SSH + IVIG	Additional IVIG combined with SSH as second-line therapy for refractory KD was safe, well-tolerated, and promising for severe cases	Ebata et al. (35)
Children with KD	Observational study	IVMP + IVIG	Strategy for refractory KD was safe and effective in preventing coronary artery aneurysm	Ebato et al. (36)
Children with KD	Observational study	Prednisolone + IVIG	Targeted use of prednisolone with the second IVIG dose for refractory KD to resist the second IVIG dose appears effective	Kimura et al. (37)
Children with KD	Observational study	IVIG	Early intravenous gamma globulin retreatment for refractory KD	Chiyonobu et al. (38)

KD, Kawasaki disease; IVIG, intravenous immunoglobulin; IVMP, intravenous methylprednisolone-pulse; SSH, sivelestat sodium hydrate.

### 4.1 Foundational treatment modalities

#### 4.1.1 High-dose IVIG

Administered at the onset of KD, high-dose IVIG constitutes the primary treatment modality, aiming to diminish the incidence of coronary artery complications (Table 1). The regimen involves a single intravenous dose of 2 grams per kilogram of body weight (27). Although the exact mechanistic action of IVIG remains partially undefined, it is postulated to attenuate the inflammatory response and modulate immune system activity (24–27).

#### 4.1.2 Aspirin therapy

Aspirin therapy in KD encompasses a dual-phased approach: initially, a high dosage is employed for its anti-inflammatory properties during the acute phase, followed by a reduced dosage post-symptom resolution to leverage its antiplatelet effects, thus preventing thrombosis (28, 29).

### 4.2 Advancements in treatment strategies

#### 4.2.1 Biologic therapy

Biologic agents, particularly anti-TNF-α therapies like infliximab, represent novel interventions for patients exhibiting resistance to IVIG (Table 1) (8, 16, 25, 30–32). These treatments have demonstrated potential in curbing inflammation and safeguarding against coronary artery damage (8, 16, 25, 30–32).

#### 4.2.2 Corticosteroid pulse therapy

For IVIG-resistant cases, high-dose corticosteroid pulse therapy, exemplified by methylprednisolone pulse therapy, offers an alternative strategy (33, 34). This approach aims for rapid mitigation of the inflammatory response, thereby decreasing the risk of coronary artery anomalies.

### 4.3 Management of refractory KD

Patients unresponsive to standard IVIG and aspirin treatment necessitate tailored therapeutic strategies. Beyond biologic and corticosteroid pulse therapies, additional immunomodulatory agents, including methotrexate and cyclophosphamide, might be considered (31, 33). Essential to the management of refractory KD is meticulous cardiovascular monitoring and, when warranted, direct coronary artery intervention (31, 33, 35–38).

In conclusion, the therapeutic landscape for KD is evolving, with innovative treatments broadening the spectrum of options for challenging cases. Validation of the efficacy and safety of these emergent strategies through comprehensive studies remains imperative. Furthermore, crafting personalized treatment protocols for KD is vital, demanding modifications tailored to the individual patient's clinical presentation and treatment responsiveness (Table 1).

## 5 Strategies for preventing cardiovascular complications

Mitigating cardiovascular complications arising from KD is pivotal for the therapeutic regimen, given the potential for these complications to profoundly affect patient health over the long term (2). Such complications predominantly entail coronary artery dilation and aneurysms (10). Effective prevention hinges on the early detection and management of predisposing factors alongside the adoption of sustained management approaches (2, 10, 39).

### 5.1 Identifying and managing risk factors for cardiovascular complications

#### 5.1.1 Risk factors

IVIG Treatment Non-Response: A lack of timely response to IVIG treatment markedly elevates the risk of coronary artery anomalies (1, 40, 41).

Elevated Inflammatory Markers: Persistently high levels of CRP and ESR are indicative of an increased complication risk (1).

Demographic Vulnerabilities: Male gender and infancy (notably, children under one year of age) are associated with heightened risk profiles (1, 40).

Prolonged Fever: An extended period of high fever during KD's progression signals a greater likelihood of coronary artery damage (41).

#### 5.1.2 Early detection methods

Echocardiography: As a cornerstone diagnostic tool, echocardiography is essential for the initial identification, post-treatment evaluation, and ongoing monitoring of coronary artery irregularities (8, 9, 41).

Laboratory Monitoring: Assessing inflammatory marker levels, such as CRP and ESR, facilitates the evaluation of treatment efficacy and cardiovascular risk (8, 9).

Clinical Vigilance: Meticulous observation of symptom evolution is critical for the early recognition of emerging cardiovascular concerns (41).

### 5.2 Strategies for long-term management to prevent cardiovascular complications

#### 5.2.1 Pharmacotherapy

Aspirin: Initiated at a high dosage for its anti-inflammatory capacity during the acute phase, followed by a maintenance low dose to exploit its antiplatelet properties, thereby thwarting thrombosis (2, 34, 42, 43).

Anticoagulation Therapy: In cases presenting coronary artery abnormalities, anticoagulants like warfarin are recommended to forestall thrombotic events (2).

#### 5.2.2 Lifestyle modifications

Routine Medical Surveillance: Incorporating echocardiography and additional cardiovascular evaluations ensures continuous health status monitoring (2, 44, 45).

Nutritional Guidelines: Advocating for a diet low in fats and abundant in fruits and vegetables diminishes cardiovascular disease risk.

Guided Physical Activity: Encouraging exercise within safe limits, under professional advisement, aids in maintaining cardiovascular health without inducing undue strain (2, 45).

Tobacco Exposure Avoidance: Steering clear of active and passive smoking is crucial, given its established risk for cardiovascular ailments (2).

In essence, a multifaceted strategy that integrates risk assessment, vigilant monitoring, pharmacological interventions, and lifestyle modifications is imperative for the prevention of cardiovascular complications in KD patients. Implementing these measures effectively minimizes complication risks and fosters a more favorable long-term health prognosis.

## 6 Future research directions

KD challenges the medical community with its unknown etiology and complex pathogenesis, particularly impacting pediatric populations. The quest for advanced treatment modalities and preventive measures against cardiovascular complications necessitates a multi-faceted research approach to enhance patient prognosis and quality of life.

### 6.1 Existing challenges

Undefined Etiology: The elusive cause of KD hampers the development of precise therapeutic interventions (2, 3, 8).

Patient Response Variability: The diverse treatment responses, especially among IVIG-resistant KD cases, underscore the need for more adaptable and effective treatment regimens (8).

Cardiovascular Risk Assessment: Current diagnostic tools, such as echocardiography, require supplementation with more precise methodologies for the early detection and accurate prediction of coronary artery anomalies (2).

### 6.2 Proposed future research directions

#### 6.2.1 Elucidating KD etiology

Etiological Investigations: Pursue research into potential infectious agents, examining the involvement of specific viruses or bacteria as triggers for KD (5, 46–48).

Genetic Studies: Investigate genetic predispositions to KD, aiming to identify relevant gene mutations and susceptibility loci, thereby illuminating genetic risk factors (46–48).

#### 6.2.2 Advancing treatment options

Targeted Therapeutic Development: Innovate drug therapies aimed at modulating the immune response or directly targeting the vasculitic processes, informed by an enriched understanding of KD's molecular underpinnings (2, 49, 50).

Biologic and Small Molecule Interventions: Evaluate the efficacy of novel anti-inflammatory and immunomodulatory agents, including targeted cytokine inhibitors and T-cell modulation strategies (2, 50).

Regenerative Medicine Applications: Assess the therapeutic potential of stem cell therapy and tissue engineering techniques in the restoration of cardiovascular integrity following KD-induced damage (49, 50).

#### 6.2.3 Preventive measures and long-term management

Risk Stratification and Early Intervention Tools: Develop and validate clinical and molecular biomarker-based risk assessment models to facilitate timely intervention and tailor treatment approaches to individual KD patient profiles (5, 51).

Lifestyle Modification Studies: Examine the influence of dietary and physical activity modifications on the prevention of KD-associated cardiovascular complications, aiming to establish evidence-based lifestyle recommendations (51).

In essence, advancing our comprehension of KD and improving patient outcomes necessitates a comprehensive research strategy encompassing basic science investigations, clinical trial innovations, and public health initiatives. Through concerted efforts across these domains, there is potential to significantly enhance the therapeutic landscape for KD, reducing the burden of complications and elevating the long-term well-being of affected individuals.

## 7 Summary

KD, characterized by systemic vasculitis primarily affecting children, lacks a clear etiology. Despite this, significant strides have been made in its diagnosis, treatment, and prevention of cardiovascular complications. Traditional therapies, such as high-dose IVIG and aspirin, effectively mitigate coronary artery abnormalities and improve short-term prognosis. Novel therapies, like biologic agents and corticosteroid pulse therapy, provide alternative options for IVIG-resistant KD cases. These advancements broaden the treatment spectrum and offer hope for better outcomes in challenging cases. Research into KD's pathogenesis reveals contributions from immune dysregulation, genetic susceptibility, and environmental factors. This understanding lays the groundwork for developing innovative diagnostic tools and treatment approaches.

The intersection of the COVID-19 pandemic and an increase in KD-like symptoms presents new challenges for research into KD's etiology and management. The association between SARS-CoV-2 and KD emphasizes the need for further studies to elucidate the mechanisms by which viral infections may precipitate or exacerbate this condition. This study not only advances our understanding of KD management but also highlights the imperative for ongoing investigations into the associations between infectious diseases and systemic vasculitides.

Despite therapeutic advancements, challenges persist in preventing and managing cardiovascular complications associated with KD. Addressing these challenges requires ongoing research efforts aimed at elucidating complex pathogenic mechanisms and developing personalized treatment strategies. Future research endeavors should focus on unraveling the intricate mechanisms underlying KD, refining treatment modalities, and tailoring interventions to individual patient needs. Through a comprehensive approach encompassing basic research, clinical trials, and patient management, the outlook for KD treatment can be significantly enhanced, leading to improved patient outcomes.
